# Complete Chloroplast Genome Sequence and Phylogenetic Analysis of *Aster tataricus*

**DOI:** 10.3390/molecules23102426

**Published:** 2018-09-21

**Authors:** Xiaofeng Shen, Shuai Guo, Yu Yin, Jingjing Zhang, Xianmei Yin, Conglian Liang, Zhangwei Wang, Bingfeng Huang, Yanhong Liu, Shuiming Xiao, Guangwei Zhu

**Affiliations:** 1 Artemisinin Research Center, Institute of Chinese Materia Medical, China Academy of Chinese Medical Sciences, Beijing 100700, China; xfshen1030@163.com; 2 College of Agricultural (College of Tree Peony), Henan University of Science and Technology, Luoyang 471023, China; shuai93guo@163.com; 3 School of Computer Science and Technology, Shandong University, Jinan 250101, China; 18384253631@163.com; 4 College of Pharmacy, Hubei University of Chinese Medicine, Wuhan 430065, China; zhangjingjing851@126.com; 5 College Pharmacy, Chengdu University of Chinese Medicine, Chengdu 611137, China; xianmeiyinCM@163.com; 6 College of Pharmacy, Shandong University of Traditional Chinese Medicine, Jinan 250355, China; conglian_liang@163.com; 7 State Key Laboratory of Innovative Natural Medicine and TCM Injections, Ganzhou 341000, China; jxwzw1@qfyy.com.cn (Z.W.); jxhbf1@qfyy.com.cn (B.H.); jxlyh1@qfyy.com.cn (Y.L.); 8 State Key Laboratory of Innovative Natural Medicine and TCM Injections, Jiangxi qingfeng pharmaceutical co. LTD, Ganzhou 341000, China

**Keywords:** *Aster tataricus*, chloroplast genome, phylogeny

## Abstract

We sequenced and analyzed the complete chloroplast genome of *Aster tataricus* (family Asteraceae), a Chinese herb used medicinally to relieve coughs and reduce sputum. The *A. tataricus* chloroplast genome was 152,992 bp in size, and harbored a pair of inverted repeat regions (IRa and IRb, each 24,850 bp) divided into a large single-copy (LSC, 84,698 bp) and a small single-copy (SSC, 18,250 bp) region. Our annotation revealed that the *A. tataricus* chloroplast genome contained 115 genes, including 81 protein-coding genes, 4 ribosomal RNA genes, and 30 transfer RNA genes. In addition, 70 simple sequence repeats (SSRs) were detected in the *A. tataricus* chloroplast genome, including mononucleotides (36), dinucleotides (1), trinucleotides (23), tetranucleotides (1), pentanucleotides (8), and hexanucleotides (1). Comparative chloroplast genome analysis of three *Aster* species indicated that a higher similarity was preserved in the IR regions than in the LSC and SSC regions, and that the differences in the degree of preservation were slighter between *A. tataricus* and *A. altaicus* than between *A. tataricus* and *A. spathulifolius*. Phylogenetic analysis revealed that *A. tataricus* was more closely related to *A. altaicus* than to *A. spathulifolius*. Our findings offer valuable information for future research on *Aster* species identification and selective breeding.

## 1. Introduction

*Aster tataricus* is a tall perennial herb of the genus *Aster* (family Asteraceae). It has been used as a therapeutic traditional medicine for eliminating phlegm and relieving cough for thousands of years [1,2], and cultivated as a high-value medicinal plant. A number of bioactive compounds have been isolated from *A. tataricus*, such as shionone, epifriedelinol, quercetin, emodin, caffeoylquinic acid, kaempferol, and some other triterpenes or saponins [3,4]. Modern pharmacological studies [5,6,7] have shown that *A. tataricus* exhibits diverse pharmacological effects, including antibacterial, antiviral, antitumor, and diuretic activities; consequently, it is recorded as a basic clinical herb in the Chinese Pharmacopoeia. Furthermore, as a late-blooming aster, *A. tataricus* is an appealing ornamental plant in some East Asian countries. However, genomic research on *A. tataricus*—both nuclear genome sequencing and plastid genome sequencing—has been relatively scarce. This lack of genetic information has hindered the basic research into, and applications of, this valuable plant, such as molecular authentication, breeding, cultivation, and bioactive compound biosynthesis.

The chloroplast, the photosynthetic organelle of most green plants, is involved in both developmental processes and secondary metabolic activities [8], as well as coordination of gene expression between organelle and nuclear genome [9]. The chloroplast (cp) genome is considered to have originated from an ancestral endosymbiotic cyanobacteria [10] and is organized into large clusters of polycistronic transcribed genes [11]. Therefore, it has a highly conserved tetrad structure, with a large single-copy (LSC) region, a small single-copy (SSC) region, and two inverse repeats (IRs) regions. As conserved matrilineal genetic information, chloroplasts play a part in cytoplasmic male sterility (CMS) [12], genetic diversity in populations [13,14,15], and especially, phylogenetic analysis [16,17]. In addition, the comparison of chloroplast sequences has been successfully used to study the evolution [18] and athentication markers to molecular identification at genus levels [19]. Evolutionary events, such as IR contraction and expansion, and re-inversion of SSC, have been found in the comparison of chloroplast genome [20,21]. Moreover, the chloroplast DNA sequences offers adequate information to study the phylogenetics and phylogeography of angiosperms at lower taxonomic levels [8]. In past decades, there have been great advances in our understanding of chloroplasts [22,23,24,25], in terms of their origin, structure, evolution, forward and reverse genetics, and genetic engineering. Moreover, the emergence of second-generation sequencing technology means that there is now less demand for research into chloroplast genomics [26,27]. Therefore, a draft cp genome assembly for *A. tataricus* is of great significance for exploring molecular identification, phylogenetic relationships, and evolutionary events studies among Asteraceae species.

Here, we reported the first whole chloroplast genome sequence for *A. tataricus*, together with the characterization of its gene annotations and repeat compositions. We also compared this chloroplast genome with that of other *Aster* species, which revealed significant variation in genome size and highly divergent regions in intergenic spacers. This comprehensive cp genomic analysis would provide a basis for molecular identification and further understanding of the evolutionary history of *Aster* species.

## 2. Results and Discussion

### 2.1. Features of A. tataricus cpDNA

The complete cp genome sequence of *A. tataricus* was 152,992 bp (GenBank accession number MH669275). The structure of the *A. tataricus* cp genome was analogous to those from other *Aster* species [28], and included an LSC region (84,698 bp; covering 55.4%), an SSC region (18,250 bp; covering 11.9%), and a pair of inverted repeats (IRA/IRB, 25,022 bp; covering 16.4%) (Table 1). The content of DNA G + C in the LSC, SSC, and IR regions, and the whole genome, was 35.2%, 31.3%, 43%, and 37.3%, respectively. The DNA G + C content is a very significant indicator when evaluating species affinity, and the cpDNA G + C content of *A. tataricus* is identical to that of other *Aster* species [28]. The DNA G + C content of the IR regions in *A. tataricus* was greater than that of other regions (LSC, SSC); this phenomenon is very common in other plants too [16,17]. The relatively high DNA G + C content of the IR regions is generally attributable to the rRNA genes and tRNA genes [29,30,31].

**Figure 1:**
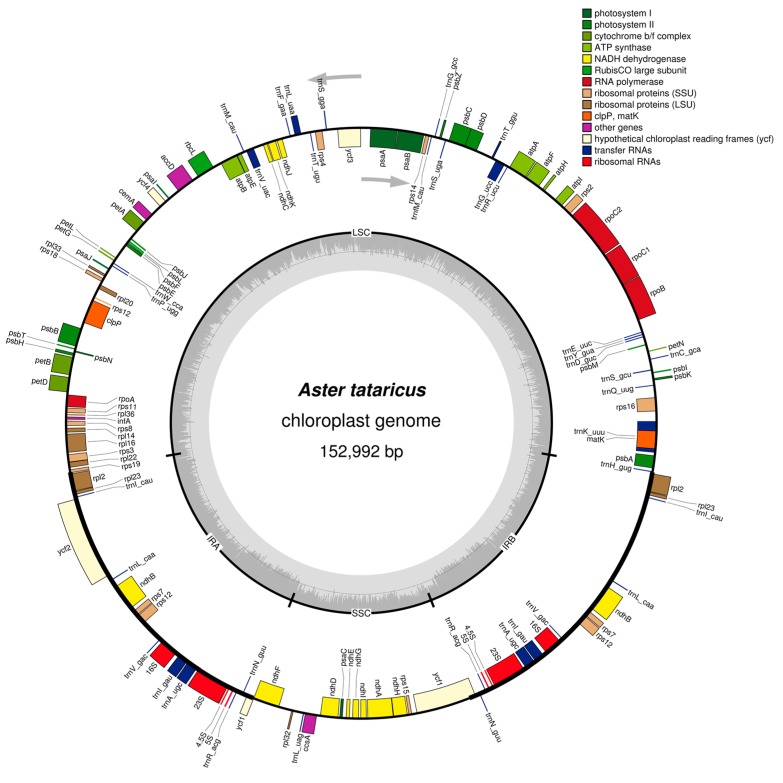
Gene map of the *A. tataricus* chloroplast genome. Genes drawn inside the circle are transcribed clockwise, and those outside are transcribed counterclockwise. Genes belonging to different functional groups are color-coded. The darker gray in the inner circle corresponds to DNA G + C content, while the lighter gray corresponds to A + T content.

In the *A. tataricus* cp genome, 115 functional genes were observed, including four rRNA genes, 30 tRNA genes, and 81 protein-coding genes (Table 2). Furthermore, 18 genes—seven tRNA, all four rRNA, and seven protein-coding genes—were repeated in the IR regions (Figure 1). The LSC region contained 62 protein-coding and 22 tRNA genes, while the SSC region comprised one tRNA gene and 12 protein-coding genes.

The sequences of the tRNA and protein-coding genes were studied, and the frequency of codon usage was inferred and summarized for the *A. tataricus* chloroplast genome. Our study revealed that 23,441 codons characterize the coding capacity of 81 protein-coding and 30 tRNA genes in *A. tataricus* (Table 3). Of these codons, 4065 (17.34%) were found to code for leucine and 204 (0.87%) for tryptophan, which represented the maximum and minimum prevalent number of amino acids in the *A. tataricus* chloroplast genome, respectively. A- and U-ending codons were ordinary.

There were 18 intron-containing genes in total: 12 protein-coding genes and six tRNA genes (Table 4). Fifteen genes (nine protein-coding and six tRNA genes) comprised one intron, and two genes (*ycf3* and *clpP*) comprised two introns (Table 4). The intron of the *trnK-UUU* gene contains the *matK* gene, and the size of the intron was 2497 bp. The *rps12* gene was a trans-spliced gene, with the 5′ end located in the LSC region and the copied 3′ end located in the IR regions. Earlier studies have reported that *ycf3* is essential for the constant accumulation of the photosystem I compound [32]. Therefore, we suppose that the intron gain in *ycf3* of *A. tataricus* may be valuable information in terms of further studies of the mechanism of photosynthesis evolution. We compared the length of exons and introns in genes with introns in the *A. tataricus* and *A. spathulifolius* chloroplast genomes (Table 4). While these were found to be broadly similar, some differences were noted: (1) in the *A. spathulifolius* chloroplast genome, the *rpl16* gene had no intron; (2) there was significant variation in *rps12* and *rps16* intron length between the two species; and (3) in *A. spathulifolius*, the *rpl12* gene had no intron.

Advances in phylogenetic research have revealed that chloroplast genome evolution encompasses both structural changes and nucleotide substitutions [33,34,35]. A few examples of these changes, including intron or gene losses [21,36], have been discovered in chloroplast genomes. Introns play an important role in regulating gene expression. They can increase gene expression at a particular position and at a specific time [37]. Intron regulation mechanisms have been reported in other species. More experimental work is required to study the relationship between intron loss and gene expression introns in *A. tataricus*.

### 2.2. Simple Sequence Repeat (SSR) Analysis

**Figure 2:**
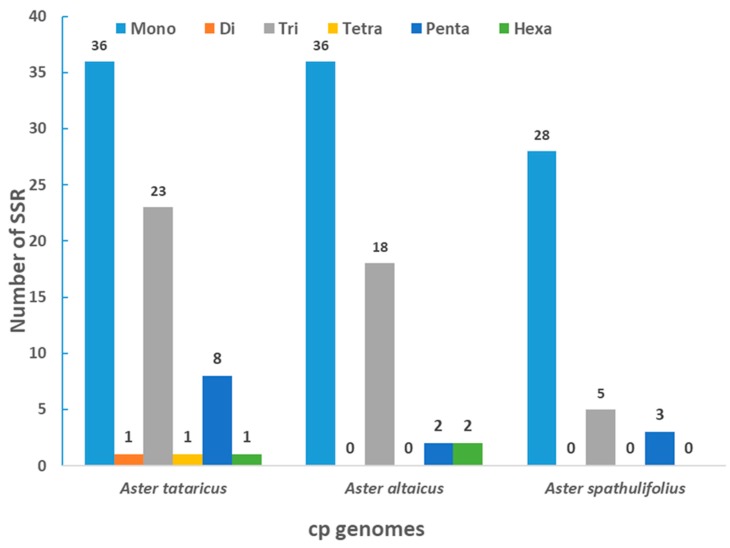
Analysis of simple sequence repeats (SSRs) in the three *Aster* chloroplast genomes.

Simple sequence repeats (SSRs) of 10 bp or longer are inclined toward slipped-strand mispairing, which is known to be the main mutational mechanism utilized in SSR polymorphisms. SSRs in the chloroplast genome can be extremely variable at the intra-specific level and are often used as genetic markers in population genetics and evolutionary studies [38,39,40,41]. In this research, we investigated the SSRs in the chloroplast genome of *A. tataricus* and in that of two other *Aster* species (Figure 2). The cp genome of *Aster tataricus*, *Aster altaicus*, and *Aster spathulifolius* contained 70, 58, and 36 SSRs, respectively. The level of mononucleotide repeat content was high (*A. tataricus*, 51.4%; *A. altaicus*, 62.1%; *A. spathulifolius*, 77.8%) in all the above species. These results will provide chloroplast SSR markers that can be used to study genetics, select germplasm for breeding, and facilitate the molecular identification of species.

### 2.3. Comparative Chloroplast Genomic Analysis

**Figure 3:**
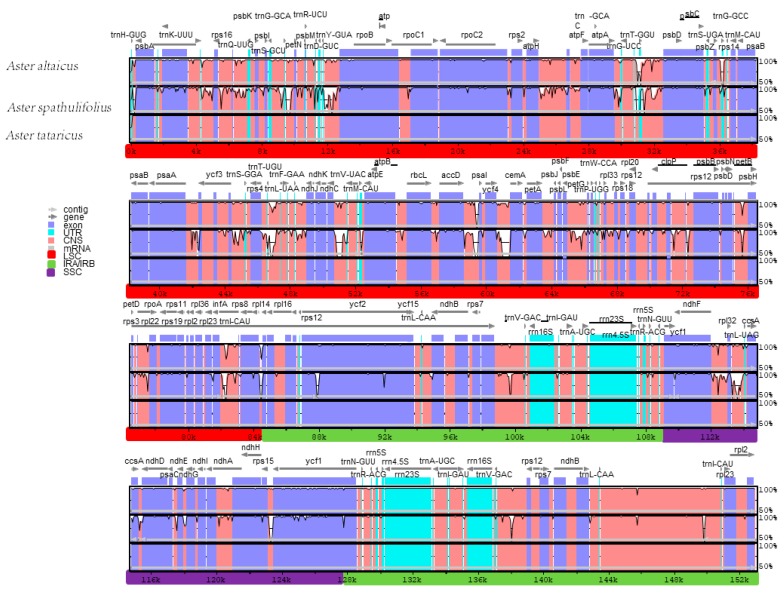
Comparison of three chloroplast genomes using mVISTA. Gray arrows and thick black lines above the alignment indicate gene orientation. Purple bars represent exons, blue bars represent untranslated regions (UTRs), pink bars represent conserved non-coding sequences (CNS), and gray bars represent mRNA. The y-axis represents the percentage identity (shown: 50–100%).

Comparative analysis of genomes is a tremendously important step in genomics [42,43]. Comparing the structural changes amongst *Aster* chloroplast genomes revealed that the chloroplast genome *A. spathulifolius* was the smallest of the three whole *Aster* chloroplast genomes (Table 1). *A. spathulifolius* had the fewest IR regions (17,973 bp) among these sequenced *Aster* chloroplast genomes. We supposed that the dissimilar length of the IR regions was the principal reason for the change in sequence length. To explicate the level of genome differences, the sequence identity of the *Aster* chloroplast DNAs was computed using mVISTA software, with *A. tataricus* as a reference (Figure 3). The results of this comparison showed that the IR (A/B) regions exhibited fewer differences than the LSC and SSC regions. Moreover, the non-coding regions showed more variability than the coding regions, and the marked differences in regions among the three chloroplast genomes were evident in the intergenic spacers. Of the three *Aster* chloroplast genomes, *A. tataricus* and *A. altaicus* exhibited the fewest differences.

### 2.4. Inverted Repeat (IR) Contraction and Expansion in the A. tataricus Chloroplast Genome

**Figure 4:**
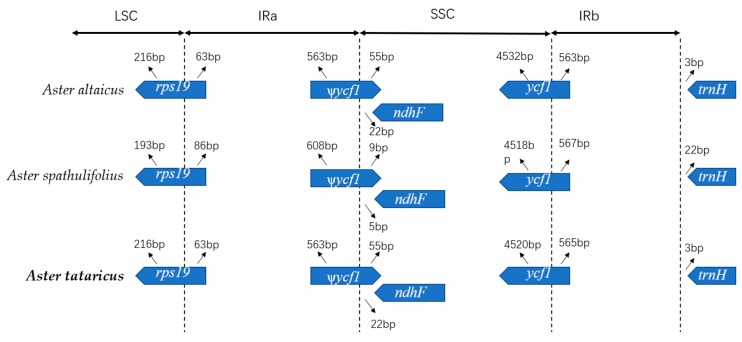
Comparison of border distance between adjacent genes and junctions of the LSC, SSC, and two IR regions among the chloroplast genomes of three *Aster* species. Boxes below the main line indicate the adjacent border genes. The figure is not to scale with respect to sequence length and only shows relative changes at or near the IR/SC borders.

Contractions and expansions of the IR regions at the borders are ordinary evolutionary events and represent the main reasons for changes in the size of chloroplast genomes; they play a significant role in evolution [44,45,46]. For *A. altaicus*, *A. spathulifolius* and *A. tataricus*, we conducted an exhaustive comparison of four junctions, LSC-IRA (JLA), LSC-IRB (JLB), SSC-IRA (JSA), and SSC-IRB (JSB), between the two IRs (IRA and IRB) and the two single-copy regions (LSC and SSC) (Figure 4). The JSA junction was placed in the *ycf1* pseudogene region in all the *Aster* species chloroplast genomes and outspread to different lengths (*A. altaicus*, 563 bp; *A. spathulifolius*, 608 bp; *A. tataricus*, 563 bp) within the IRA region of all the genomes; the IRB region contained 563, 567, and 565 bp of the *ycf1* gene, respectively. Recently, it was reported that *ycf1* is required for plant viability and codes Tic214, a significant component of the *Arabidopsis*Tic complex member [47,48]. Correspondingly, the *trnH* gene was placed in the LSC region, 3, 22, and 3 bp away from the IRB/LSC border in the three *Aster* chloroplast genomes, respectively. The JLA in the *Aster* species was overlapped by *rps19*. The *ndhF* gene was found to be 22, 5, and 22 bp away from the IRA/SSC border in the *Aster* species.

Although the gene order in chloroplasts is generally conserved in most green plants, it has been reported that many sequences are rearranged in chloroplast genomes from an extensive variety of different plant species, including inversions in the LSC region, IR contractions or expansions with inversions, and re-inversion in the SSC region [49,50,51,52,53]. Sequence rearrangements that convert chloroplast genome structure in related species may also reveal information about genetic diversity that could be used for molecular classification and evolution studies.

### 2.5. Phylogenetic Analysis 

**Figure 5:**
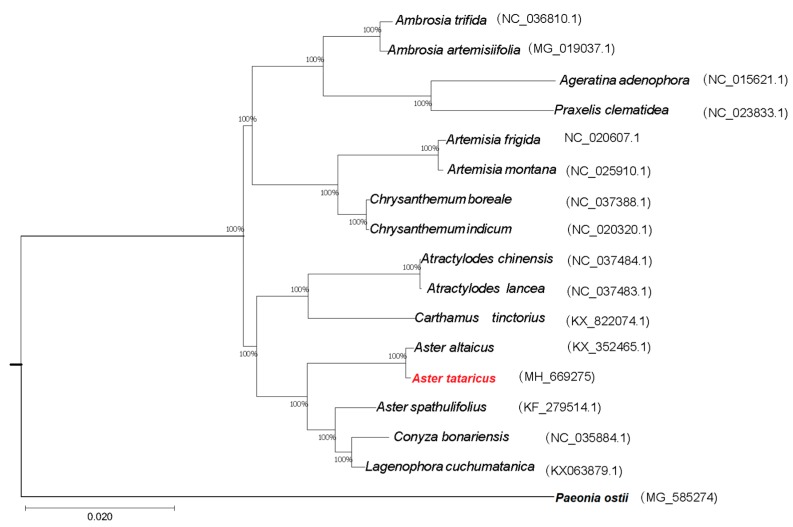
Maximum likelihood (ML) phylogenetic tree reconstruction including 17 species based on concatenated sequences from all chloroplast genomes. The position of *A. tataricus* is indicated in red text. *Paeonia ostii* was used as the outgroup.

The availability of a completed *A. tataricus* cp genome provided us with sequence information that can be used to study the phylogeny of *A. tataricus* among Asteraceae. We performed multiple sequence alignments using the whole cp genome sequences in 16 Asteraceae species. One additional cp genome, *Paeonia ostii* (Paeoniaceae), was included as an outgroup (Figure 5). The method of maximum likelihood (ML) was used to construct a phylogenetic tree. The results strongly supported the finding that *A. altaicus* and *A. tataricus* are sister species, and *A. tataricus* is closer to *A. altaicus* than to *A. spathulifolius*.

## 3. Materials and Methods

### 3.1. DNA Sequencing, Chloroplast Genome Assembly, and Validation 

The *A. tataricus* was planted in the China Academy of Chinese Medical Sciences (N 39°56′, E 116°25′, Beijing, China). Fresh leaves were gathered and covered with tin foil, frozen in liquid nitrogen, and maintained at −80 °C. An improved cetyltrimethylammonium bromide (CTAB) method was used to obtain the whole genomic DNA of *A. tataricus* [54]. The concentration of DNA was estimated using an ND-2000 spectrometer (Nanodrop Technologies, Wilmington, DE, USA) [55]. A 250-bp shotgun library was constructed according to the manufacturer’s instructions (Vazyme Biotech Co. Ltd., Nanjing, China). The library was sequenced using an Illumina X Ten platform (Illumina, San Diego, CA, USA) double terminal sequencing method (150 pair-ends). The sample contained 5 G of raw data, and over 34 million paired-end reads (SRA accession: SRP154896) were obtained. 

The raw data was filtered using Skewer-0.2.2 (Institute of Plant Quarantine Research, Chinese Academy of Inspection and Quarantine, Beijing, China, https://sourceforge.net/projects/skewer/) [56]. BLAST searches were used to abstract chloroplast-like reads from clean-reads in comparison with reference sequences (*A. altaicus*). Lastly, we used the chloroplast-like reads to assemble sequences using SOAPdenovo-2.04 (BGI·tech, Shenzhen, China, https://sourceforge.net/projects/soapdenovo2/files/SOAPdenovo2/) [57]. SSPACE-3.0 (Leiden University, Leiden, The Netherlands https://www.baseclear.com/services/bioinformatics/basetools/sspace-standard/) [58] and GapCloser-1.12 (BGI·tech, Shenzhen, China, https://sourceforge.net/projects/ oapdenovo2/files/GapCloser/) [59] were used to outspread sequences and fill gaps. PCR amplification and Sanger sequencing were used to confirm the four junction regions between the IR regions and the LSC/SSC regions, to confirm the assembly (Table S1).

### 3.2. Gene Annotation and Sequence Analyses

CpGAVAS [60] was used to annotate the sequences; DOGMA [61] and BLAST were used to check the annotation findings. tRNAscanSEv1.21 [62], with default settings, was used to identify all tRNA genes. OGDRAWv1.2 [63] was used to show the structural features of the chloroplast genomes. Relative synonymous codon usage (RSCU) values were defined using MEGA5.2 [64].

### 3.3. Genome Comparison

mVISTA [65] (Shuffle-LAGAN mode) was used to compare the whole chloroplast genome of *A. tataricus*, *A. altaicus* (KX352465), and *A. spathulifolius* (KF279514), with the annotation of *A. tataricus* as the reference. Phobos version 3.3.12 [66] was employed to detect SSRs within the cp genome, with the search parameters set at 10 repeat units for mononucleotides, _8 repeat units for dinucleotides,_4 repeat units for trinucleotides and tetranucleotides, and _3 repeat units for pentanucleotide and hexanucleotide SSRs. 

### 3.4. Phylogenetic Analysis 

We downloaded 16 whole chloroplast genome sequences of Asteraceae species from the National Center for Biotechnology Information (NCBI) Organelle Genome and Nucleotide Resources database. The whole chloroplast genome sequences were used to analyze the phylogenetics. The software clustalw2 (The Conway Institute of Biomolecular and Biomedical Research, Dublin, Ireland) was used to align sequences. MEGA5.2 was used to analyze and plot the phylogenetic tree with ML (maximum likelihood). We used 1000 replicates and TBR (tree bisection and reconnection) branch exchange to complete the bootstrap analysis. Furthermore, *Paeonia ostii* was set as the outgroup.

## 4. Conclusions

To our knowledge, we were the first to complete the sequencing and analysis of the whole chloroplast genome of *A. tataricus*, showing that the quadruple structure, gene order, DNA G + C content, and codon usage features were similar to those of the other *Aster* chloroplast genomes studied. Compared with the chloroplast genomes of the other two *Aster* species, the chloroplast genome of *A. tataricus* was the largest, while the genome structure and composition were found to be similar. Of the three *Aster* chloroplast genomes, *A. tataricus* and *A. altaicus* exhibited the fewest differences. Examination of the phylogenetic relationships among the three *Aster* species revealed that *A. tataricus* was more closely related to *A. altaicus* than to *A. spathulifolius*. The findings of this study offer an assembly of a whole chloroplast genome of *A. tataricus*, which would be valuable for molecular identification, breeding, and further biological discoveries.

## Figures and Tables

**Table 1:** Summary of complete chloroplast genomes for three *Aster* species.

Species	*Aster altaicus*	*Aster spathulifolius*	*Aster tataricus*
**Large single-copy (LSC)**			
Length (bp)	84,240	81,998	84,698
G + C (%)	35.3	31.4	35.2
Length (%)	55.3	54.9	55.4
**Small single-copy (SSC)**			
Length (bp)	18,196	17,973	18,250
G + C (%)	31.3	35.8	31.3
Length (%)	11.9	12.0	11.9
**IR**			
Length (bp)	25,005	24,751	25,022
G + C (%)	43.0	43.2	43.0
Length (%)	16.4	16.6	16.4
**Total**			
Length (bp)	152,446	149,473	152,992
G + C (%)	37.3	37.7	37.3

**Table 2:** Genes in the *A. tataricus* chloroplast genome.

Category	Gene Group	Gene Names
Self-replication	Large subunit of ribosomal proteins	rpl2 **^,a^, *14*, *16* **, *20*, *22*, *23* ^a^, *32*, *33*, *36*
	Small subunit of ribosomal proteins	*rps2*, *3*, *4*, *7*^a^, *8*, *11*, *12* **^,a^, *14*, *16* **, *18*, *19*
	DNA-dependent RNA polymerase	*rpoA*, *B*, *C1* **, *C2*
	rRNA genes	*rrn16S*^a^, *rrn23S*^a^, *rrn4.5S*^a^, *rrn5S*^a^
	tRNA genes	*trnA-UGC* **^,a^, *trnC-GCA*, *trnD-GUC*, *trnE-UUC*, *trnF-GAA*, *trnfM-CAU*, *trnG-UCC* **, *trnG-GCC, trnH-GUG, trnI-CAU, trnI-GAU* **^,a^, *trnK-UUU* **, *trnL-CAA*, *trnL-UAA* **, *trnL-UAG*, *trnM-CAU*, *trnN-GUU*, *trnP-UGG*, *trnQ-UUG*, *trnR-ACG*, *trnR-UCU*, *trnS-GCU*, *trnS-GGA*, *trnS-UGA, trnT-GGU*, *trnT-UGU*, *trnV-GAC*, *trnV-UAC* **, *trnW-CCA*, *trnY-GUA*
Photosynthesis	Photosystem I	*psaA*, *B*, *C*, *I*, *J*
	Photosystem II	*psbA*, *B*, *C*, *D*, *E*, *F*, *H*, *I*, *J*, *K*, *L*, *M*, *N*, *T*, *Z*,
	NADH oxidoreductase	ndhA **, B **^,a^*, C*, *D*, *E*, *F*, *G*, *H*, *I*, *J*, *K*
	Cytochrome b6/f complex	petA, B **, D **, *G*, *L*, *N*
	ATP synthase	*atpA, B, E, F* **, *H, I*
	Rubisco	*rbcL*
Other genes	Maturase	*matK*
	Protease	clpP **
	Envelope membrane protein	*cemA*
	Subunit acetyl-CoA-carboxylase	*AccD*
	c-Type cytochrome synthesis gene	*CcsA*
	Conserved open reading frames	*ycf1*, *2*^a^, *3* **, *4*, *15*

** Genes containing introns. ^a^ Duplicated gene (genes present in the inverted repeat (IR) regions).

**Table 3:** Codon–anticodon recognition patterns and codon usage for the *A. tataricus* chloroplast genome.

Amino Acid	Codon	No.	RSCU *	tRNA	Amino Acid	Codon	No.	RSCU *	tRNA
Phe	UUU	1064	1.34		Tyr	UAU	793	1.39	
Phe	UUC	528	0.66	*trnF-GAA*	Tyr	UAC	348	0.61	*trnY-GUA*
Leu	UUA	554	1.55	*trnL-UAA*	Stop	UAA	508	1.09	
Leu	UUG	492	1.37	*trnL-CAA*	Stop	UAG	368	0.79	
Leu	CUU	459	1.28		His	CAU	374	1.36	
Leu	CUC	205	0.57		His	CAC	175	0.64	*trnH-GUG*
Leu	CUA	272	0.76	*trnL-UAG*	Gln	CAA	482	1.4	*trnQ-UUG*
Leu	CUG	169	0.47		Gln	CAG	206	0.6	
Ile	AUU	798	1.46		Asn	AAU	792	1.39	
Ile	AUC	397	0.73	*trnI-GAU*	Asn	AAC	347	0.61	*trnN-GUU*
Ile	AUA	441	0.81	*trnI-UAU*	Lys	AAA	914	1.42	*trnK-UUU*
Met	AUG	415	1	*trn(f)M-CAU*	Lys	AAG	376	0.58	
Val	GUU	364	1.45		Asp	GAU	471	1.43	
Val	GUC	176	0.7	*trnV-GAC*	Asp	GAC	187	0.57	*trnD-GUC*
Val	GUA	318	1.26	*trnV-UAC*	Glu	GAA	550	1.39	*trnE-UUC*
Val	GUG	148	0.59		Glu	GAG	241	0.61	
Ser	UCU	509	1.37		Cys	UGU	345	1.15	
Ser	UCC	329	0.89	*trnS-GGA*	Cys	UGC	253	0.85	*trnC-GCA*
Ser	UCA	493	1.33	*trnS-UGA*	Stop	UGA	524	1.12	
Ser	UCG	292	0.79		Trp	UGG	491	1	*trnW-CCA*
Pro	CCU	259	1.29		Arg	CGU	204	0.73	*trnR-ACG*
Pro	CCC	156	0.78	*trnP-GGG*	Arg	CGC	108	0.39	
Pro	CCA	224	1.12	*trnP-UGG*	Arg	CGA	282	1.01	
Pro	CCG	164	0.82		Arg	CGG	173	0.62	
Thr	ACU	321	1.22		Arg	AGA	329	0.89	*trnR-UCU*
Thr	ACC	246	0.93	*trnT-GGU*	Arg	AGG	277	0.75	
Thr	ACA	314	1.19	*trnT-UGU*	Ser	AGU	578	2.06	
Thr	ACG	174	0.66		Ser	AGC	337	1.2	*trnS-GCU*
Ala	GCU	250	1.25		Gly	GGU	315	0.95	
Ala	GCC	169	0.85		Gly	GGC	205	0.62	*trnG-GCC*
Ala	GCA	242	1.21	*trnA-UGC*	Gly	GGA	466	1.4	*trnG-UCC*
Ala	GCG	138	0.69		Gly	GGG	342	1.03	

* RSCU: relative synonymous codon usage.

**Table 4:** A comparison of exon and intron length in genes with introns in the *A. tataricus* and *A. spathulifolius* chloroplast genomes.

Gene	Location	Exon I (bp)	Intron I (bp)	Exon II (bp)	Intron II (bp)	Exon III (bp)
*trnK-UUU*	LSC	37	2497	38		
		37	2502	35		
*trnG-UCC*	LSC	23	732	48		
		23	723	47		
*trnL-UAA*	LSC	34	441	50		
		37	423	50		
*trnV-UAC*	LSC	36	575	37		
		38	573	37		
*trnI-GAU*	IR	38	781	35		
		43	776	35		
*trnA-UGC*	IR	38	820	35		
		38	820	35		
*rps12* *	LSC	234	535	25	——	114
		114	——	243	——	243
*rps16*	LSC	234	820	40		
		39	826	216		
*rpl16*	LSC	402	1008	10		
		——	——	——		
*rpl2*	IR	391	671	434		
		393	668	435		
*rpoC1*	LSC	431	709	1639		
		429	721	1641		
*ndhA*	SSC	552	1055	540		
		553	1105	540		
*ndhB*	IR	777	674	756		
		777	670	756		
*ycf3*	LSC	124	690	228	739	155
		124	697	230	739	153
*petB*	LSC	6	754	658		
		6	745	642		
*atpF*	LSC	144	718	411		
		145	699	410		
*clpP*	LSC	71	812	291	614	229
		71	800	291	623	229
*petD*	LSC	9	645	526		
		9	724	474		

Exon and intron lengths in genes with introns in the *A. tataricus* chloroplast genome (gray background), and in the *A. spathulifolius* chloroplast genome (normal background) * The *rps12* gene is a trans-spliced gene with the 5′ end located in the LSC region and the duplicated 3′ ends located in the IR regions.

## Supplementary Materials

The following are available online. Table S1. Primers used for assembly validation.
