# Supplementary material for: Complete Chloroplast Genome Sequence and Phylogenetic Analysis of Aster tataricus
Source: Molecules. 2018 Sep 21;23(10):2426. doi: 10.3390/molecules23102426 (PMC6222381; doi:10.3390/molecules23102426)
Supplement: Supplementary file 1 [file molecules-23-02426-s001.pdf]

Table S1. Primers used for assembly validation.

Primer	Sequence (5’>3’)	Amplicon Size (bp)
LSC_IRa	TTGCTTCATTCGAACCCATA<br>CGGCCACACTATTGCTATCC	515
IRa_SSC	TTACGGGACAGCTTCGATCT<br>TTACGGGACAGCTTCGATCT	529
SSC_IRb	CGCTTCTTACGGAGCCTTTA<br>TGCGTAATCTCAGCATTCAA	645
IRb_LSC	ATGTTGGGGTGAACCAGAAA<br>CGTGCTAACCTTGGTATGGAA	711
